# Characterizing epistatic hotspots of human disease

**DOI:** 10.1186/1753-6561-6-S6-O12

**Published:** 2012-10-01

**Authors:** Tallulah Andrews, Caleb Webber

**Affiliations:** 1 MRC Functional Genomics Unit, Department of Physiology Anatomy and Genetics, University of Oxford, Oxford, Oxfordshire OX1 3PT, UK

## Background

We recently reported that single *de novo* copy number variants (CNVs) in patients with developmental disorders frequently affected multiple functionally similar genes [1]. Since functional clusters are present in many eukaryotic species, including humans [2], a single CNV can affect multiple genes, potentially incurring additional deleterious effects due to epistatic interactions. Furthermore, the position of genes within a biological pathway may be relevant to the phenotypic impact of their disruption [3]. In this study, we investigated the roles that epistatic interactions and the network position of disrupted genes play in developmental disorders.

## Materials and methods

We obtained a set of 626 *de novo* CNVs identified in patients with developmental disorders from the DatabasE of Chromosomal Imbalances and Phenotype in Humans using Ensemble Resources (DECIPHER). Employing a novel integrated functional linkage network, we examined clustering and importance (centrality within the network) of the genes affected by these *de novo* CNVs. A fast neighbor joining clustering algorithm was used to identify gene groups within each CNV defined as those linked by the top 1 % of shortest paths within the functional linkage network. To ensure functional clusters were not simply the result of tandem duplications, we collapsed paralogous genes into a single copy within the genome. Significant enrichments in clustering and central network position were used to build a predictive model able to scan the genome and identify similar regions whose copy number change may also predispose to disease.

## Results

DECIPHER *de novo* CNVs were significantly enriched for large functional clusters with low within-cluster similarity compared with gene-number matched randomizations. Functional clusters were even more significantly enriched when paralogous genes were collapsed. Clusters were present in 357 of 626 CNVs, with an average size of four genes. In addition all measures of network centrality were significantly high, with average maximum betweenness (bottlenecks) the most significant. Bottleneck genes tended to be haplo-insufficient and highly pleiotropic when knocked out in mice. Functional clusters frequently contained bottleneck genes and these regions were frequently affected by CNVs in more than one patient.

## Conclusions

DECIPHER *de novo* CNVs identify putative epistatic hotspots, which are clusters of functionally related genes whose disruptions are associated with developmental disorders. In addition, these hotspots are enriched in bottleneck genes that may play a role in the diversity of phenotypes observed for these patients.

## References

[B1] Boulding H Webber C Large-scale objective association of mouse phenotypes with human symptoms through structural variation identified in patients with developmental disorders Hum Mutat 2012 33 874 883 10.1002/humu.22069 22396327

[B2] Al-Shahrour F Minguez P Margues-Bonet T Gazave E Navarro A Dopazo J Selection upon genome architecture: Conservation of functional neighborhoods with changing genes PLoS Comput Biol 2010 6 e1000953 10.1371/journal.pcbi.1000953 20949098 PMC2951340

[B3] Yu H Kim PM Sprecher E Trifonov V Gerstein M The importance of bottlenecks in protein networks: Correlation with gene essentiality and expression dynamics PLoS Comput Biol 2007 3 e59 10.1371/journal.pcbi.0030059 17447836 PMC1853125

